# Immunological dynamics of memory T cells in gram-negative septic shock patients: a prospective clinical study

**DOI:** 10.3389/fimmu.2026.1923966

**Published:** 2026-09-17

**Authors:** Shuhang Wang, Li Liu, Ruichen Mao, Yanfen Chai, Yancun Liu

**Affiliations:** Tianjin Medical University General Hospital, Tianjin, China

**Keywords:** clinical prognosis, gram-negative septic shock, immune dysfunction, immunology, memory T cells

## Abstract

**Background:**

Septic shock induced by Gram-negative bacilli can lead to severe immune dysfunction. The dysfunction and reprogramming of memory T cells are closely involved in the immunosuppressive state of septic shock. This study aimed to investigate the dynamic changes of memory T cells in such patients and their association with clinical outcomes.

**Methods:**

This study is a prospective clinical research. Thirty-five patients with Gram-negative septic shock were enrolled and classified into survival and non-survival groups. Peripheral blood samples were obtained on admission, day 2 and day 7 after admission, and patients were stratified into survival and death groups based on 28-day survival status. Flow cytometry was performed to analyze the proportions of lymphocyte subsets as well as CD4^+^ and CD8^+^ memory T cell subsets, including naive, effector, central memory, and effector memory T cells. Intracellular cytokine staining was used to evaluate their functional capacity to IL-2, TNF-α, and IFN-γ.

**Results:**

Compared with survivors, non-survivors showed significantly reduced counts of CD8^+^ T cells, B cells, and NK cells. In memory T-cell subset analysis, non-survivors exhibited a significantly higher proportion of CD8^+^ T_CM_ cells at admission and a markedly lower proportion of CD8^+^ T_EM_ cells on day 7. Functionally, CD8^+^ T_CM_ cells from non-survivors showed enhanced IL-2 production, while CD8^+^ T_EM_ cells displayed diminished TNF-α and IFN-γ production on day 7. Further analysis revealed that the proportion and activation status of CD8^+^ T_CM_ cells at admission had good predictive value for 28-day mortality.

**Conclusion:**

Patients with Gram-negative septic shock exhibited early elevation in the proportion and activity of CD8^+^ T_CM_ cells, and a decrease in the number and functional impairment of CD8^+^ T_EM_ cells in later stages. The dynamic changes in CD8^+^ memory T-cell subsets are associated with clinical outcomes, showing preliminary potential as candidate immune indicators for prognosis assessment, which needs further validation in larger cohorts.

## 1 Introduction

Sepsis is a life-threatening organ dysfunction caused by a dysregulated host response to infection. Its complex pathophysiology involves an excessive inflammatory response followed by an immunosuppressive state (1). This immune system dysfunction is a key contributor to the high mortality rate and susceptibility to secondary infections in patients (2, 3). Although the overactivation of the innate immune system has garnered significant attention in the early phases of sepsis, growing evidence indicates that the adaptive immune system, particularly T lymphocytes responsible for long-term immune surveillance and memory, is also profoundly affected during the course of sepsis and is closely associated with poor prognosis (4, 5).

Memory T cells are central to maintaining long-term immune protection in the adaptive immune system. Through their unique immune memory mechanisms, they can rapidly mount a highly effective immune response when the body encounters a reinfection, thereby effectively controlling the reactivation of latent viruses and preventing secondary infections (6). Based on differences in the expression of the homing receptor CCR7 and the differentiation antigen CD45RA (7), memory T cells can be further classified into several functional subsets, including central memory, effector memory, and stem cell-like memory T cells (8). Each subset plays a distinct role in immune surveillance, activation, migration, and effector functions; among these, memory CD8^+^ T cells are a key subset involved in controlling the reactivation of latent viruses and defending against secondary infections (9). However, sepsis, as a severe systemic inflammatory response syndrome, has been confirmed by existing clinical observations and basic research to lead to a decrease in the number and dysfunction of CD4^+^ and CD8^+^ T lymphocytes, especially severely impairing the immune surveillance function of memory CD8^+^T cells (10, 11). This damage further leads to reactivation of latent viruses, exacerbates immune disorders in sepsis patients, and affects clinical prognosis, ultimately significantly altering the phenotype and functional homeostasis of the memory T cell population (12, 13). Although previous studies have examined the immune status of patients with sepsis (14, 15), further research is needed to investigate the dynamic changes in memory T cell subsets during the course of the disease and their association with patient prognosis in the clinical context of septic shock caused by Gram-negative bacilli.

Therefore, this study aims to conduct longitudinal sampling and analysis in patients with septic shock. It will systematically examine the dynamic changes in the frequencies of various memory T cell subsets in peripheral blood and their cytokine production capacities. Furthermore, it seeks to explore the relationship between these immunological parameters and the 28-day clinical prognosis of the patients, providing a new perspective for a deeper understanding of the immunopathological mechanism of septic shock and potential basis for exploring prognostic biomarkers based on immunophenotypes.

## 2 Methods and materials

### 2.1 Study subjects

This study is a prospective clinical research. Patients with Gram-negative bacilli-induced septic shock admitted to the Emergency Intensive Care Unit (EICU) of Tianjin Medical University General Hospital between March 2021 and September 2022 were enrolled. The primary endpoint was 28-day all-cause mortality. Secondary clinical endpoints included length of hospital stay, ICU stay, duration of mechanical ventilation, duration of antibiotic therapy, and the occurrence of nosocomial or opportunistic infections. All participants or their legal guardians provided written informed consent. The study was approved by the Medical Ethics Committee of Tianjin Medical University General Hospital (IRB2021-YX-220-01) and registered at the Chinese Clinical Trial Registry (Principal Investigator: Liu Yancun; Registration Number: ChiCTR2200060826).

A total of 35 patients were included in this study, and the inclusion process is shown in **Figure 1**. Inclusion Criteria: (1) Meeting the diagnostic criteria for septic shock according to the Sepsis-3 international consensus definitions: serum lactate level >2 mmol/L (18 mg/dL) and the requirement for vasopressor therapy to maintain a mean arterial pressure (MAP) ≥65 mmHg despite adequate fluid resuscitation; (2) Age between 18 and 80 years; (3) Etiological evidence confirming Gram-negative bacterial infection. Exclusion Criteria: (1) History of severe immunodeficiency (e.g., malignancy, HIV infection, hematological diseases) or long-term use of corticosteroids or immunosuppressive drugs; (2) Patients with notifiable infectious diseases classified as Category A, B, or C in China; (3) Pregnant or postpartum women; (4) Incomplete clinical data.

**Figure 1:**
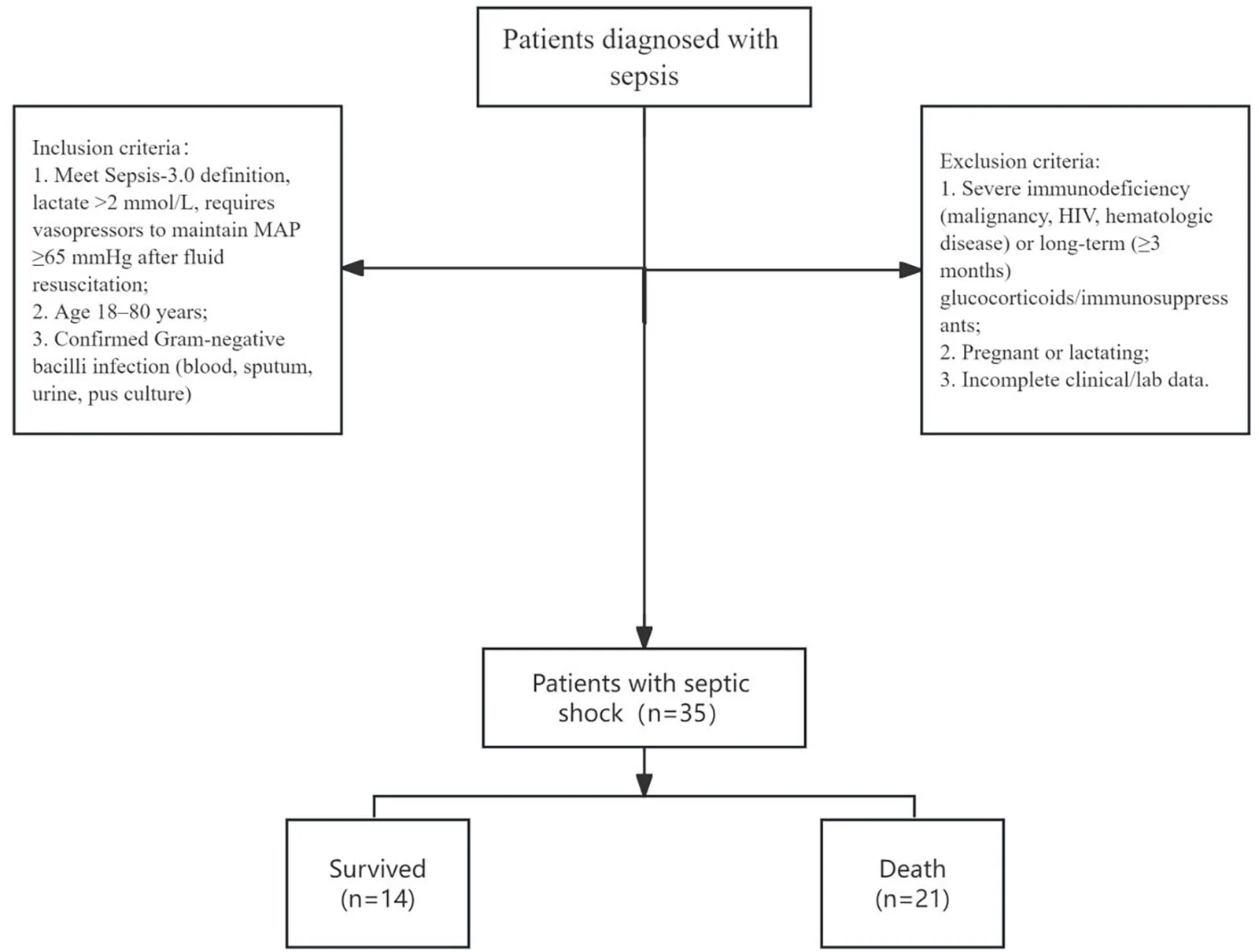
Flow chart.

### 2.2 Clinical data collection

Collected data from enrolled subjects comprised baseline characteristics including sex, age and comorbidities; primary site of infection such as bloodstream, pulmonary, urinary tract and abdominal infection as well as corresponding etiological evidence; organ function indicators including total bilirubin (TBIL), serum creatinine, norepinephrine (NE) dosage, oxygenation index (OI), Glasgow Coma Scale (GCS) score, platelet count, platelet distribution width (PDW), serum lactate level and Sequential Organ Failure Assessment (SOFA) score; and infection-related biomarkers including total white blood cell (WBC) count, differential leukocyte count, C-reactive protein (CRP) and procalcitonin (PCT).

Upon enrollment, patient vital signs and relevant laboratory results were recorded immediately, and peripheral blood samples were collected for lymphocyte subset analysis, memory T cell phenotyping, and intracellular cytokine staining (ICCS) by flow cytometry. Follow-up peripheral blood testing and clinical data collection were performed on hospital days 2 and 7. The study conducted a 28 day follow-up to record the results of the primary and secondary outcomes.

### 2.3 Flow cytometric analysis of immune cell phenotype

Peripheral blood samples were collected at admission and on days 2 and 7 using EDTA anticoagulant tubes and processed within 24 hours. For immunophenotyping of major lymphocyte subsets, 50 μL of anticoagulated whole blood was incubated with 20 μL of a fluorescently labeled monoclonal antibody cocktail (CD3-FITC, CD16+CD56-PE, CD45-PerCP, CD19-APC, Tongsheng Shidai Bio-Technology Co., Ltd.) in a flow cytometry tube protected from light. Red blood cells were then lysed, absolute counting beads were added, and samples were analyzed by flow cytometry. For memory T cell subset characterization, 200 μL of whole blood was processed with red blood cell lysis buffer, washed, and stained with fluorescent antibodies againstCD3-APC-cy7, CCR7-APC, CD8-PE-cy7, CD4-PerCP, CD45RA-FITC, CD45RO-PE (BioLegend). Samples were analyzed on a Beckman Coulter DxFLEX flow cytometer. Naive (T_naive_), effector (T_eff_), central memory (T_CM_), effector memory (T_EM_) and stem cell-like memory T cells (T_SCM_) subsets were defined based on surface marker expression patterns. Gating strategies for CD8^+^ and CD4^+^ memory T-cell subsets followed identical hierarchical principles. First, lymphocytes were selected based on forward and side scatter; then CD3^+^ T cells were gated. Within CD3^+^ cells, CD8^+^ and CD4^+^ populations were separately identified and further stratified by CCR7 expression into CCR7^+^ and CCR7^-^ compartments. For CD8^+^ memory T cells, the CCR7^+^ population was subdivided by CD45RA and CD45RO into T_Naive_ (CD3^+^CD8^+^CCR7^+^CD45RA^+^CD45RO^-^), T_CM_ (CD3^+^CD8^+^CCR7^+^CD45RA^-^CD45RO^+^), and T_SCM_ (CD3^+^CD8^+^CCR7^+^CD45RA^+^CD45RO^+^); the CCR7^-^ population was subdivided into T_eff_ (CD3^+^CD8^+^CCR7^-^CD45RA^+^CD45RO^-^) and T_EM_ (CD3^+^CD8^+^CCR7^-^CD45RA^-^CD45RO^+^). For CD4^+^ memory T cells, the same approach was applied. For intracellular cytokine assays, parallel unstimulated control samples were run to define background fluorescence threshold. Peripheral blood lymphocyte subset analysis was conducted by flow cytometry on a Beckman Coulter DxFLEX flow cytometer (Beckman Coulter, Brea, CA, USA).

### 2.4 Detection of cytokines using flow cytometry and enzyme-linked immunosorbent assay

For functional assessment, 400 μL of whole blood was co-cultured for 6 hours in a stimulation system containing phorbol 12-myristate 13-acetate (PMA) and ionomycin, with a protein transport inhibitor added. After surface marker staining, cells were fixed, permeabilized, and intracellularly stained with antibodies directed against target cytokines.IL-2 production was assessed in CD8^+^ T_CM_ cells, while TNF-α and IFN-γ production were assessed in CD8^+^ T_EM_ cells, consistent with the classical paradigm established by Sallusto et al. (16, 17). Finally, cells were resuspended in FACS buffer for flow cytometric analysis.

Plasma levels of cytokines were quantified using ELISA. Plasma was separated by centrifugation and stored frozen. During analysis, samples and serially diluted standards were added to the plate, followed by incubation, washing, enzyme-conjugated antibody addition, substrate development, and absorbance measurement at 450 nm. Concentrations were calculated based on the standard curve.

### 2.5 Statistical analysis

Receiver operating characteristic (ROC) curves were constructed to evaluate the predictive value of CD8^+^ memory T-cell subsets and their functional parameters for 28-day mortality, with calculation of area under the curve (AUC), 95% CI, sensitivity, specificity, and optimal cutoff values by Youden index. Kaplan-Meier method was used for survival analysis, and differences between groups were compared by log-rank test.

Statistical analysis was performed using SPSS software (version 26.0) and GraphPad Prism (version 9.0). For longitudinal measurements collected at admission, day 2 and day 7, between-group comparisons were performed separately at each time point. This strategy was adopted to account for non-random missing data caused by patient mortality and maintain statistical robustness given the small sample size. The normality of continuous variables was assessed using the Shapiro-Wilk test. Normally distributed data are presented as mean ± standard deviation (mean ± SD) and were compared between groups using independent-samples t-test. Non-normally distributed data are presented as the median with interquartile range (IQR: P25, P75) and were compared using the Wilcoxon rank-sum test. Categorical variables are presented as percentage and were compared using the Chi-square test or Fisher’s exact test, as appropriate. A two-sided p-value < 0.05 was considered statistically significant.

## 3 Results

### 3.1 Baseline features

Patients were stratified into survivor and non-survivor groups based on 28-day outcomes. Compared with survivors, non-survivors exhibited a more pronounced immunosuppressive state, with significantly reduced lymphocyte count (0.58 ± 0.43 vs 1.18 ± 0.44 ×10^9^/L, P < 0.001), CD8^+^ T cell count (225.70 ± 166.10 vs 384.30 ± 242.20/μL, P = 0.041), B cell count (99.00 ± 86.88 vs 209.40 ± 133.00/μL, P = 0.010), and NK cell count (85.29 ± 88.87 vs 174.70 ± 98.94/μL, P = 0.010). Concurrently, non-survivors demonstrated more severe organ dysfunction, as evidenced by significantly higher serum creatinine levels (250.10 ± 233.70 vs 133.30 ± 88.68 μmol/L, P = 0.045), norepinephrine dosage (1.19 ± 0.60 vs 0.47 ± 0.21 μg/kg/min, P < 0.001), SOFA score (13.43 ± 4.33 vs 9.33 ± 3.07, P = 0.003), and lactate levels (8.13 ± 1.71 vs 4.29 ± 1.15 mmol/L, P < 0.001). In contrast, the OI (105.70 ± 42.50 vs 149.90 ± 51.54, P = 0.012) and GCS score (10.50 ± 3.57 vs 13.86 ± 1.77, P = 0.001) were significantly lower in non-survivors. No statistically significant differences were observed between the two groups in WBC count, neutrophil count, monocyte count, CD4^+^ T cell count, platelet count, PDW, PCT, and CRP levels. There were also no notable intergroup differences in the distribution of primary infection sites.

### 3.2 Clinical outcomes

A total of 35 patients diagnosed with septic shock were enrolled in this study. Among them, 21 were survivors and 14 were non-survivors, yielding an overall 28-day mortality rate of 40.0%. This finding is consistent with the substantial mortality burden attributable to septic shock. Among non-survivors, early mortality occurred in 28.6% of cases, with the remaining 10 patients dying between day 3 and day 28, highlighting the rapid and protracted fatal courses seen in septic shock.

Analysis of infection sources identified pulmonary infection as the most common site (40.0%), followed by urinary tract infection (28.6%), bloodstream infection (17.1%), and liver abscess (8.6%). Microbiological evaluation confirmed Gram-negative bacteria as the primary pathogens, consistent with known etiological patterns. Escherichia coli (28.6%) and other Gram-negative species (42.9%) were most frequent, followed by Proteus species (17.1%) and Klebsiella pneumoniae (11.4%). No statistically significant differences were observed between survivor and non-survivor groups regarding age, gender, and these microbiological characteristics (P > 0.05).

### 3.3 The association between lymphocyte subsets and prognosis in patients with septic shock

Peripheral blood samples obtained at hospital admission were analyzed for lymphocyte subsets using flow cytometry, with the detailed gating strategy shown in **Figure 2a**. As presented in **Table 1** and **Figure 2b**, the CD4^+^ T cell count showed no statistically significant difference between non-survivors and survivors (**Supplementary Figure S1**). In contrast, non-survivors exhibited significantly lower counts of CD8^+^ T cells, NK cells, and B cells compared to survivors. This pattern of selective lymphopenia in non-survivors is consistent with previous clinical observations in septic shock populations (18).

**Figure 2:**
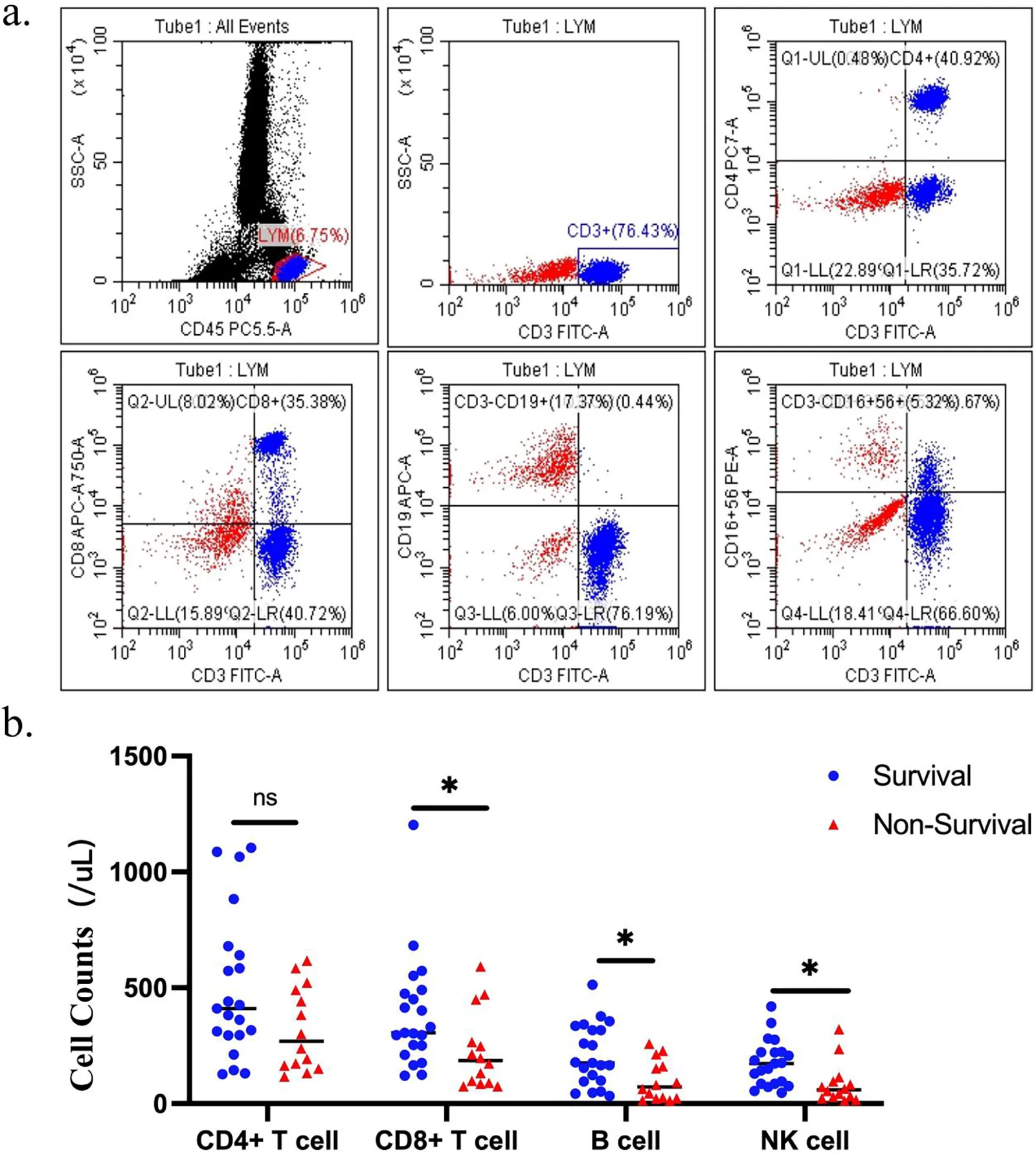
**(a)** Flow cytometric analysis of peripheral blood lymphocyte subsets; **(b)** Lymphocyte subsets in survivors and non-survivors of septic shock at admission. Between-group comparisons were performed using the independent-samples t-test for normally distributed data, and the Wilcoxon rank-sum test for non-normally distributed data. *P < 0.05, ns: not statistically significant.

**Table 1:** Baseline characteristics and clinical parameters.

Patients’ characteristics	Total (n=35)	Survivors (n=21)	Non-survivors(n=14)	P value
Age (mean± SD)	66.86 ± 16.94	67.05 ± 15.06	66.57 ± 20.04	0.937
Male, n (%)	21 (60.00)	11 (52.38)	10 (71.43)	0.311
WBC (/pL) (mean± SD)	13.17 ± 8.59	14.71 ± 9.88	10.86 ± 5.77	0.198
Lymphocytes (/pL) (mean± SD)	0.94 ± 0.52	1.18 ± 0.44	0.58 ± 0.43	<0.001
Neutrophils (/pL) (mean± SD)	11.56 ± 8.24	12.82 ± 9.62	9.67 ± 5.35	0.273
Monocytes (/pL) (mean± SD)	0.67 ± 0.45	0.71 ± 0.36	0.61 ± 0.58	0.536
CD4^+^ T cells (/uL) (mean± SD)	427.90 ± 277.10	498.50 ± 310.40	321.90 ± 180.00	0.064
CD8^+^ T cells (/uL) (mean± SD)	320.90 ± 226.40	384.30 ± 242.20	225.70 ± 166.10	0.041
B cells (/uL) (mean± SD)	165.30 ± 127.70	209.40 ± 133.00	99.00 ± 86.88	0.010
NK cells (/uL) (mean± SD)	138.90 ± 103.70	174.70 ± 98.94	85.29 ± 88.87	0.010
Platelet (/pL) (mean± SD)	158.60 ± 73.54	165.60 ± 67.73	148.10 ± 82.99	0.499
PDW (mean± SD)	13.41 ± 3.17	13.33 ± 3.48	13.54 ± 2.75	0.853
TBIL (umol/L) (mean± SD)	18.41 ± 15.53	17.32 ± 15.74	20.04 ± 15.66	0.620
Creatine (umol/L) (mean± SD)	180.10 ± 170.0	133.30 ± 88.68	250.10 ± 233.70	0.045
NE dose (ug/Kg/min) (mean± SD)	0.76 ± 0.54	0.47 ± 0.21	1.19 ± 0.60	<0.001
OI (mean± SD)	132.20 ± 52.29	149.90 ± 51.54	105.70 ± 42.50	0.012
GCS (mean± SD)	12.51 ± 3.08	13.86 ± 1.77	10.50 ± 3.57	0.001
SOFA (mean± SD)	10.97 ± 4.11	9.33 ± 3.07	13.43 ± 4.33	0.003
Lactate (mmol/L) (mean± SD)	5.82 ± 2.35	4.29 ± 1.15	8.13 ± 1.71	<0.001
PCT (ng/ml) (mean± SD)	23.18 ± 19.17	23.85 ± 20.87	22.17 ± 17.01	0.803
CRP (mg/L) (mean± SD)	116.60 ± 64.06	115.40 ± 66.80	118.40 ± 62.14	0.895
Infection sites				
Blood, n (%)	6 (17.14)	2 (9.52)	4 (28.57)	0.191
Lung, n (%)	16 (45.71)	9 (42.86)	7 (50.00)	0.739
Liver, n (%)	3 (8.57)	2 (9.52)	1 (7.14)	0.999
Urinary tract, n (%)	10 (28.57)	8 (38.10)	2 (14.29)	0.151
Bacteria				
*Escherichia coli*, n (%)	10 (28.57)	4 (19.05)	6 (42.86)	0.151
*Klebsiella pneumoniae*, n (%)	4 (11.43)	3 (14.29)	1 (7.14)	0.635
*Proteus*, n (%)	6 (17.14)	5 (23.81)	1 (7.14)	0.366
Others, n (%)	15 (42.86)	9 (42.86)	6 (42.86)	0.999

### 3.4 Dynamic changes in memory T cells in patients with septic shock

Flow cytometric analysis of surface markers on memory T cells was performed on peripheral blood samples collected at different time points (**Figures 3a, b**), allowing for observation of the dynamic changes in memory T cell subsets during the course of septic shock. Longitudinal analysis revealed specific temporal patterns within the CD8^+^ memory T cell compartment. The proportion of CD8^+^ T_CM_ increased during the early phase of the illness and subsequently declined as the disease progressed. In contrast, CD8^+^ T_EM_ showed a progressive decreasing trend in the later stages of the disease. CD8^+^ T_eff_ exhibited a slow initial increase before returning to baseline levels with disease progression, while the proportion of CD8^+^ T_SCM_ remained relatively stable throughout the observation period.

**Figure 3:**
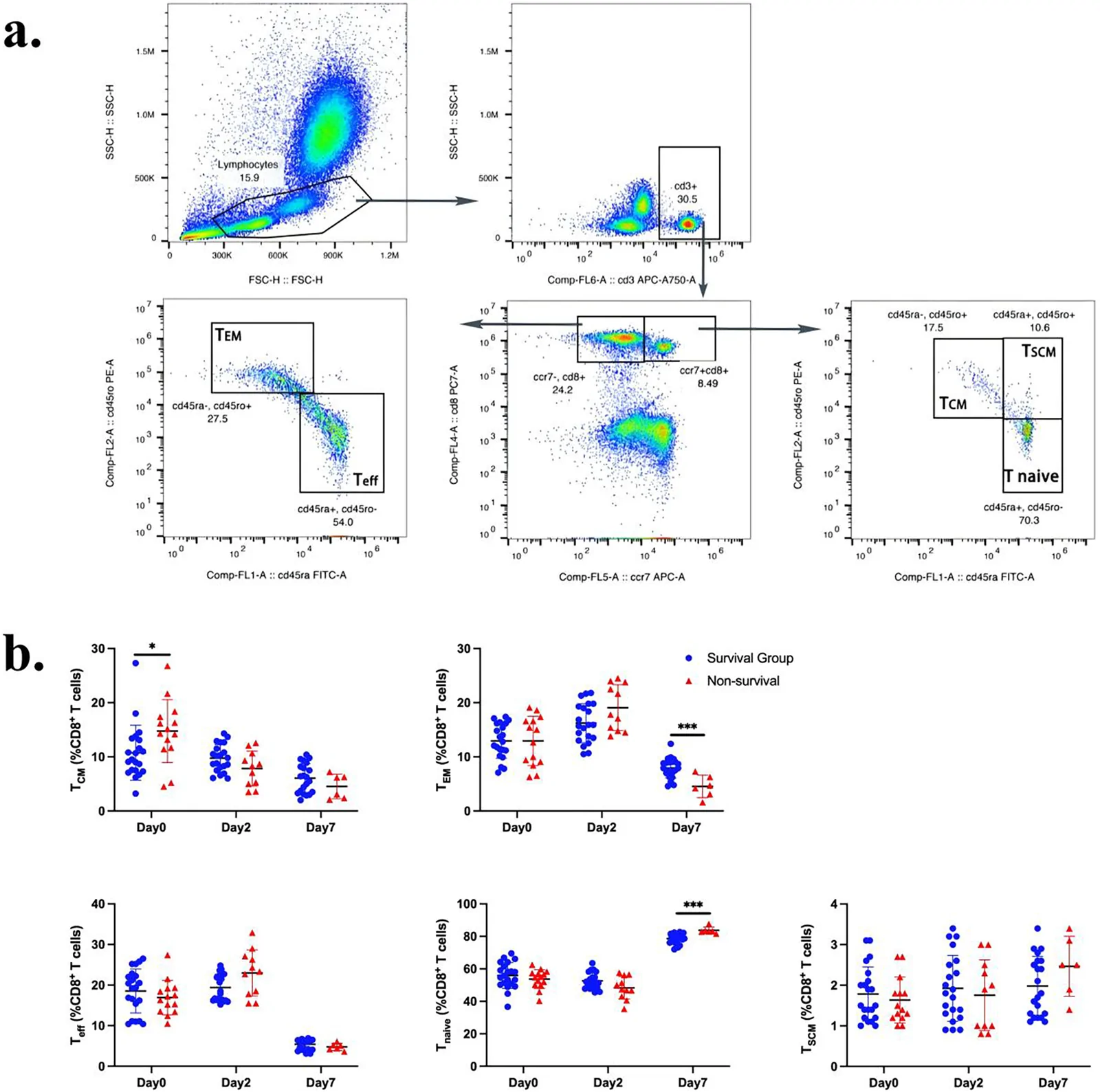
**(a)** Hierarchical gating strategy for identifying CD8^+^ memory T cell subsets; **(b)** Dynamic changes in CD8^+^ memory T cell subsets between survivors and non-survivors of septic shock. Between-group comparisons were performed using the independent-samples t-test for normally distributed data, and the Wilcoxon rank-sum test for non-normally distributed data. *P < 0.05, ns: not statistically significant.

Comparative analysis between survivors and non-survivors revealed significant differences in the distribution of memory T cell subsets. At the time of admission, non-survivors had a significantly higher proportion of CD8^+^ T_CM_ compared to survivors. By day 7, non-survivors exhibited a markedly lower proportion of CD8^+^ T_EM_ alongside a corresponding increase in the proportion of CD8^+^ T_naive_ (**Figure 3b**). No statistically significant differences were observed between the two groups for the other CD8^+^ memory T cell subsets. Furthermore, no significant differences were found between survivors and non-survivors in any of the CD4^+^ memory T cell subsets across all time points. Non-survivors with septic shock were characterized by a higher proportion of CD8^+^ T_CM_ at admission and a lower proportion of CD8^+^ T_EM_ compared with survivors.

### 3.5 Functional impairment of CD8^+^ memory T cells in septic shock patients

To further evaluate the functional status of CD8^+^ memory T cells, intracellular cytokine staining followed by flow cytometric analysis was performed on peripheral blood mononuclear cells from the patients. **Figure 4** revealed significant functional differences between non-survivors and survivors. At admission, non-survivors not only exhibited a higher proportion of CD8^+^ T_CM_ cells but also showed a more pronounced state of activation in this subset. Following stimulation with PMA and ionomycin, CD8^+^ T_CM_ cells from non-survivors demonstrated a significantly enhanced capacity to produce IL-2 compared to those from survivors, suggesting a heightened early pro-inflammatory response.

**Figure 4:**
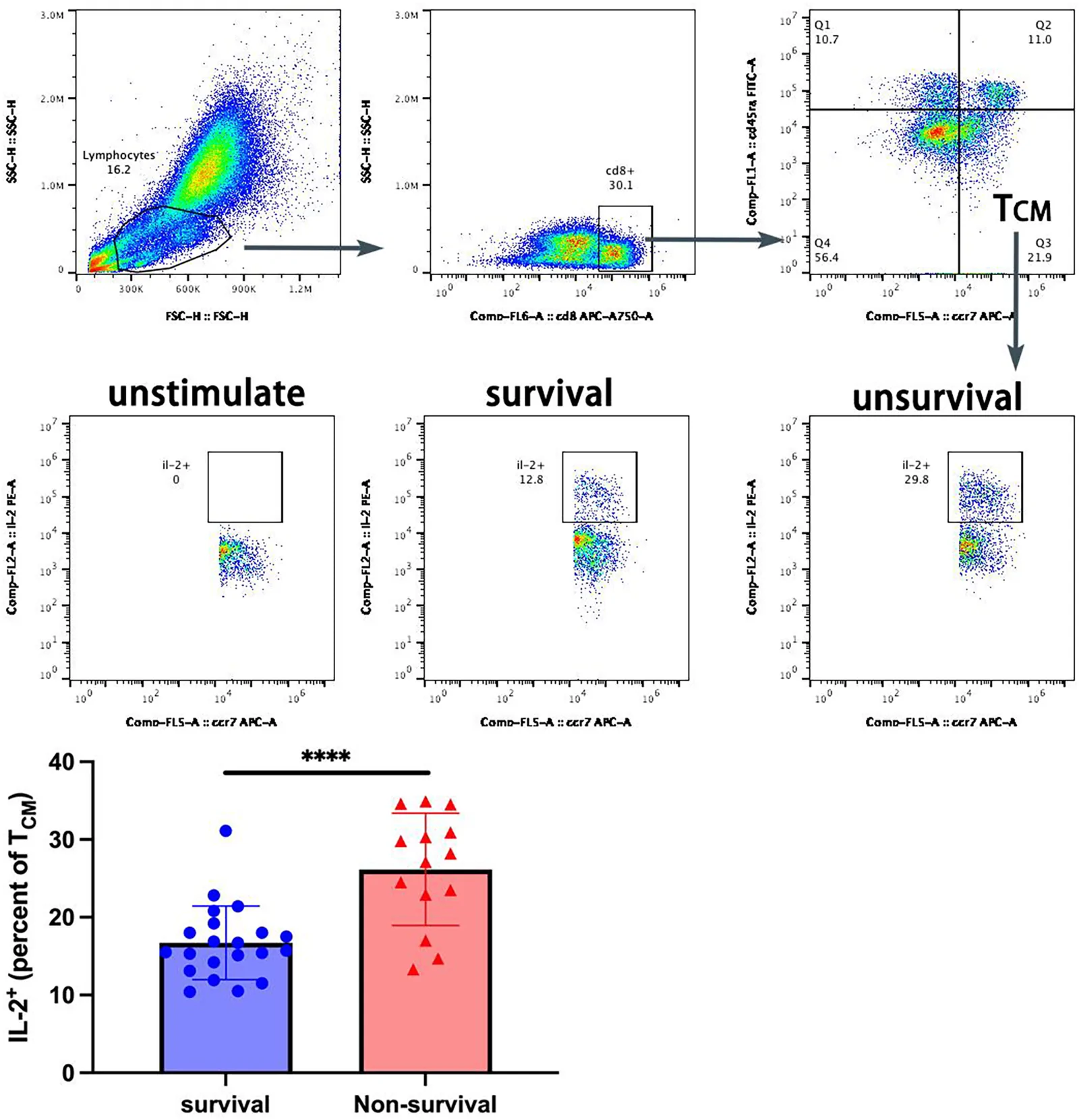
Enhanced activation of TCM at admission in non-survivors of septic shock. Between-group comparisons were performed using the independent-samples t-test for normally distributed data, and the Wilcoxon rank-sum test for non-normally distributed data. *P < 0.05, ns: not statistically significant.

By hospital day 7, the proportion of CD8^+^ T_EM_ cells in non-survivors decreased, accompanied by significant impairment of their effector functions. Under the same stimulatory conditions, the ability of CD8^+^ T_EM_ cells from non-survivors to produce TNF-α and IFN-γ was significantly lower than that of survivors, indicating an immunosuppressive state that may predispose these patients to secondary infections (**Figure 5**). At admission, CD8^+^ T_CM_ cells from non-survivors showed enhanced IL-2 production capacity. On day 7, CD8^+^ T_EM_ cells from non-survivors exhibited significantly reduced TNF-α and IFN-γ production compared with survivors.

**Figure 5:**
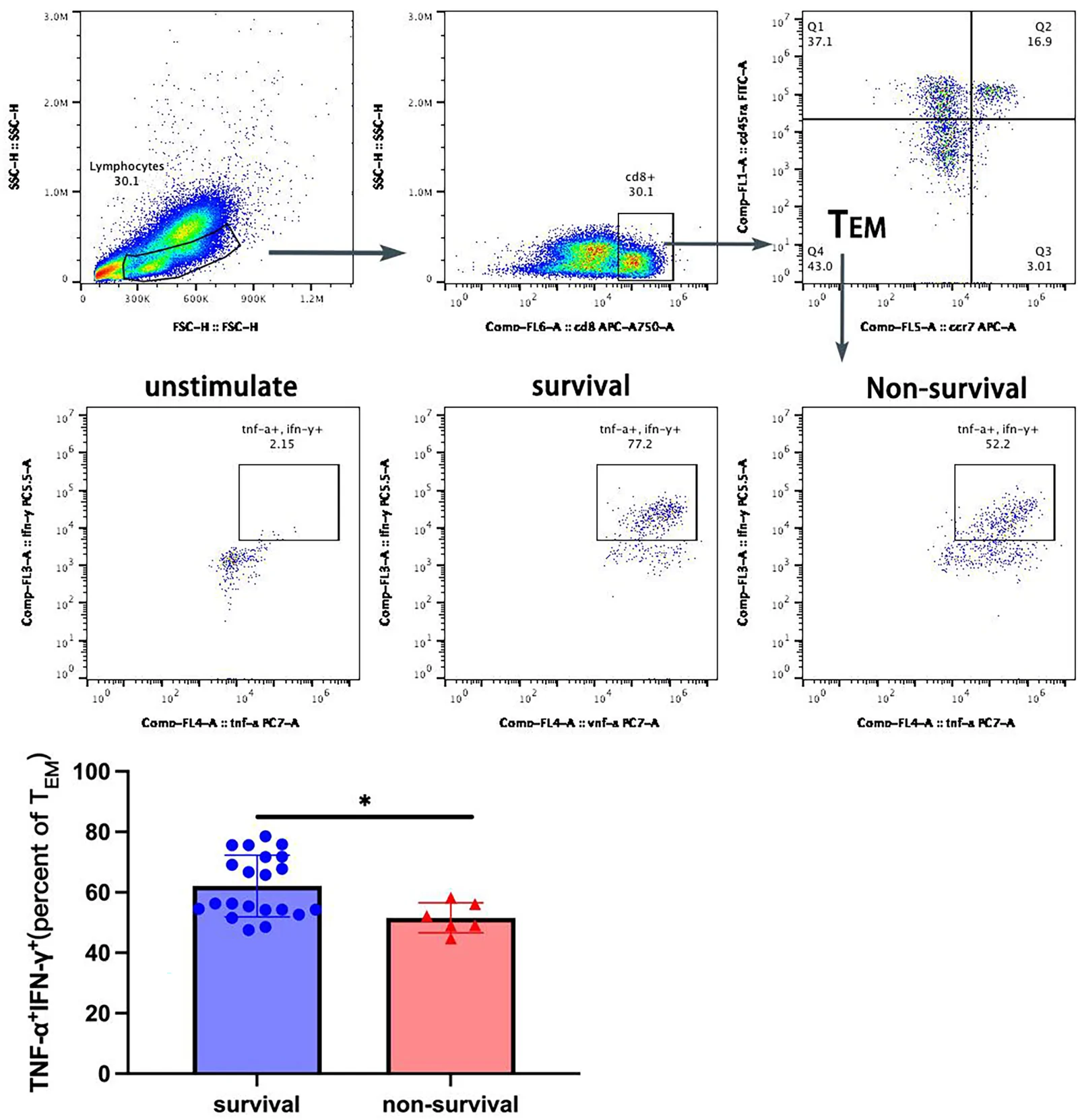
Functional impairment of T_EM_ on day 7 after admission in non-survivors of septic shock. Between-group comparisons were performed using the independent-samples t-test for normally distributed data, and the Wilcoxon rank-sum test for non-normally distributed data. *P < 0.05, ns: not statistically significant.

Additionally, ELISA was used to measure cytokine levels in the peripheral blood of patients (**Supplementary Figure S2**). At admission, there were no significant differences in plasma IL-2 levels between the two groups. At day 7, there were no significant differences in plasma TNF-α levels between the two groups; however, patients in the survival group exhibited significantly higher plasma IFN-γ levels than those in the non-survival group.

### 3.6 Prognostic value of CD8^+^ memory T cells in septic shock patients

To explore the association between CD8+ memory T cells and their functional status and 28-day mortality, we performed ROC curve analysis to assess their predictive performance in this exploratory cohort. The results demonstrated that multiple CD8^+^ memory T cell parameters at different time points possessed good predictive value. The proportion of CD8^+^ T_CM_ cells at admission yielded an AUC of 0.745 (95% CI: 0.56-0.93, P = 0.015), with a cutoff value of 11.20% corresponding to a sensitivity of 85.71% and a specificity of 66.67%. The functional activity of CD8^+^ T_CM_ cells (IL-2^+^ CD8^+^ T_CM_) at admission demonstrated even higher predictive power, with an AUC of 0.840 (95% CI: 0.69-0.99, P < 0.001). A cutoff of 22.85% provided a sensitivity of 78.57% and a specificity of 95.24% (**Figure 6**). Parameters measured on day 7 also showed significant predictive value. The proportion of CD8^+^ T_EM_ cells achieved an AUC of 0.901 (95% CI: 0.77-1.00, P = 0.003), with a cutoff of 7.4% yielding 100% sensitivity and 66.67% specificity. The functional activity of CD8^+^ T_EM_ cells (TNF-α^+^IFN-γ^+^ CD8^+^ T_EM_) produced an AUC of 0.786 (95% CI: 0.59-0.98, P = 0.036), with a cutoff of 53.40% corresponding to a sensitivity of 66.67% and a specificity of 80.95%.

**Figure 6:**
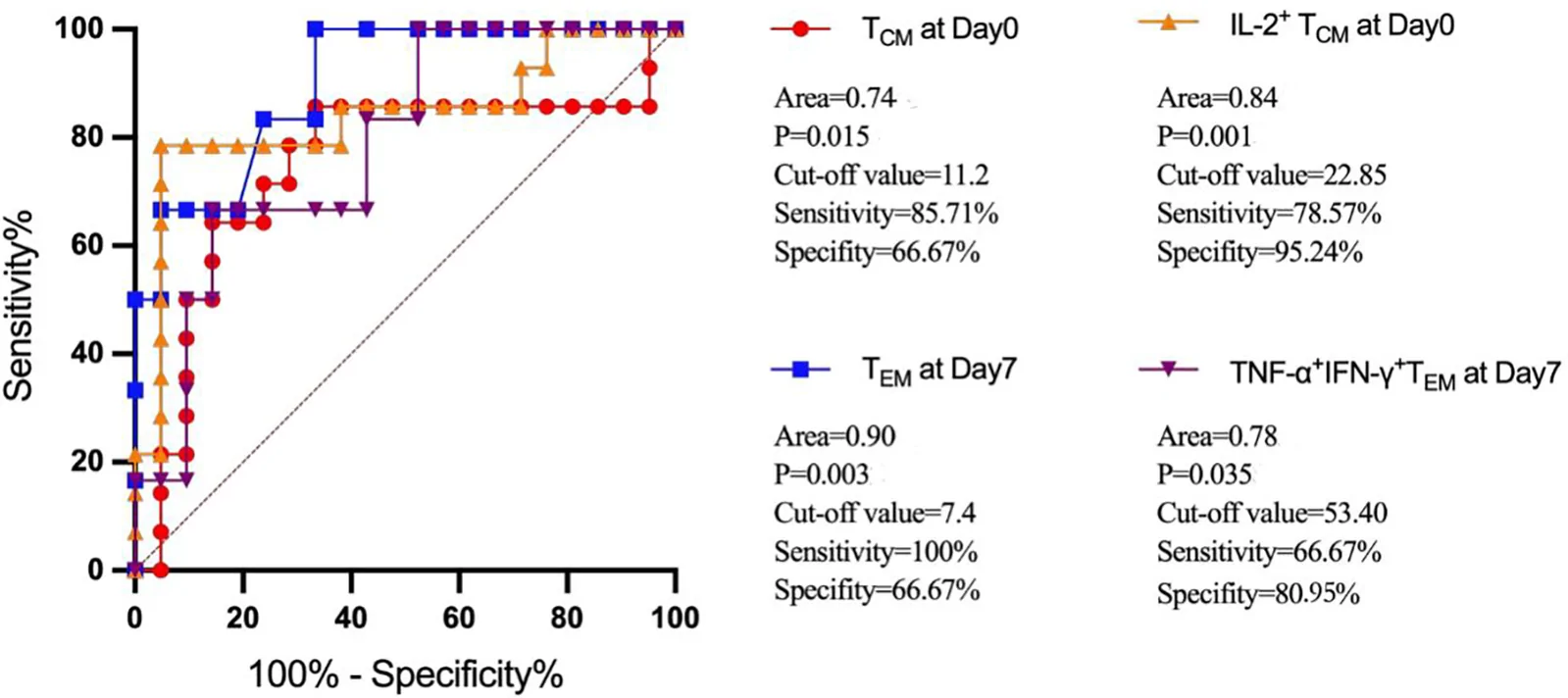
Prognostic value of CD8^+^ TCM at admission and CD8^+^ TEM on day 7 for 28-day mortality in septic shock patients. ROC curve analysis was performed, and optimal cutoff values were determined by the Youden index.

Based on the cutoff values determined by ROC analysis, we further conducted survival analysis. Using the admission CD8^+^ T_CM_ proportion cutoff of 11.2%, patients were stratified into high and low proportion groups. KM analysis revealed a significant difference in 28-day mortality between these groups (P = 0.002), with a high hazard ratio of 7.24 (95% CI: 2.53-20.75) (**Figure 7a**). Similarly, when patients were grouped using the admission IL-2^+^ CD8^+^ T_CM_ proportion cutoff of 22.85%, a significant difference in 28-day mortality was observed between the high and low groups (P < 0.001), with a hazard ratio of 11.58 (95% CI: 3.40-39.43) (**Figure 7b**).

**Figure 7:**
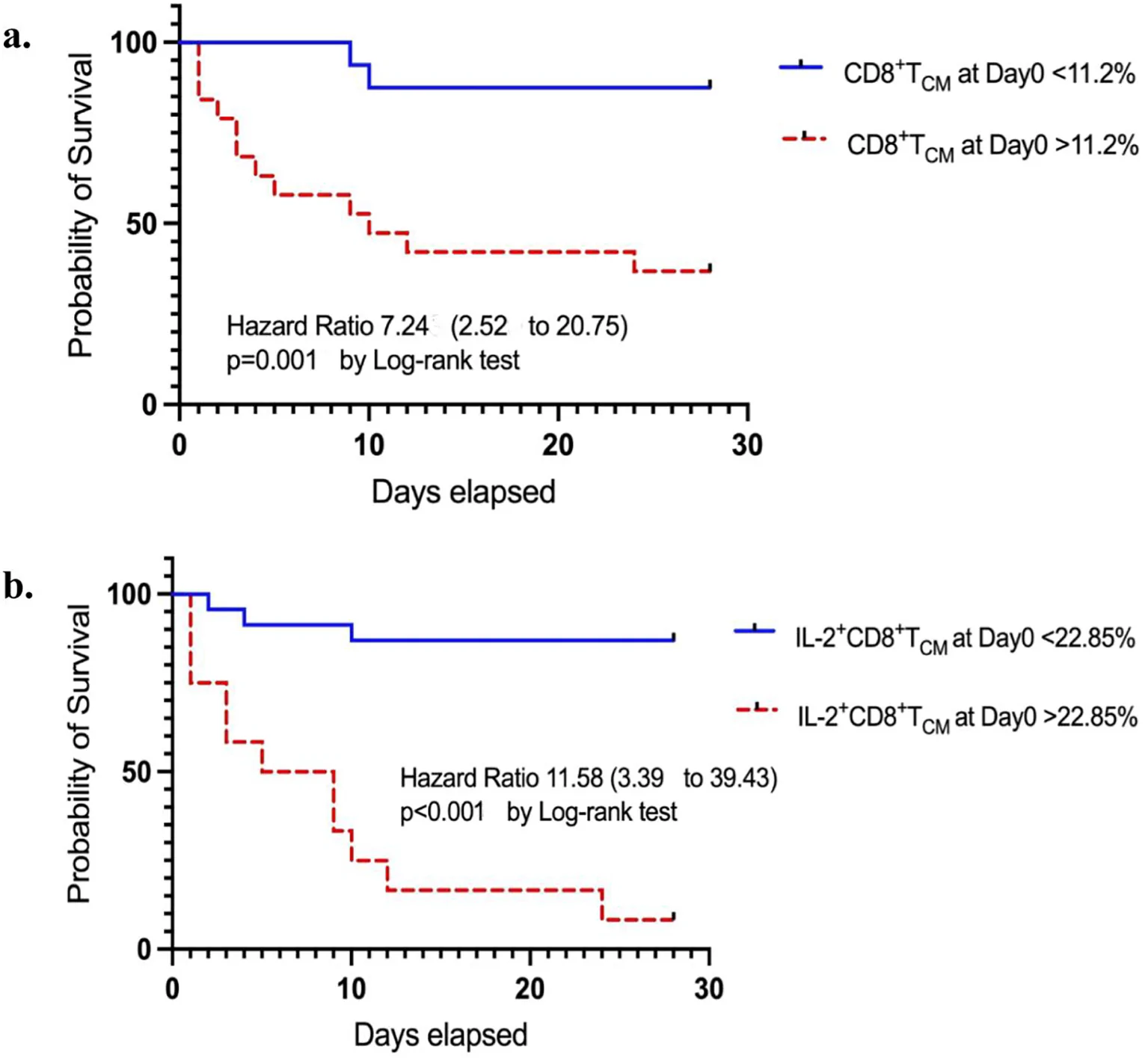
**(a)** KM survival analysis of septic shock patients stratified by admission peripheral blood CD8^+^ TCM proportion; **(b)** KM survival analysis of septic shock patients stratified by activation proportion of CD8^+^ TCM (IL-2^+^CD8^+^ TCM) at admission. Between-group survival differences were compared by the log-rank test, and hazard ratios were calculated via univariable Cox proportional hazards regression.

These exploratory results suggest that both the early proportion and functional status of CD8^+^ T_CM_ cells, as well as the later proportion and functional status of CD8^+^ T_EM_ cells, are associated with 28-day mortality in this cohort, and may provide preliminary reference for prognostic evaluation. These findings provide additional observational evidence supporting the potential value of immune cell monitoring in the prognostic assessment of septic shock.

## 4 Discussion

This prospective study provides a systematic characterization of the dynamic changes in memory T cell subsets and their functions in patients with Gram-negative bacilli-induced septic shock. Our results demonstrate that distinct CD8^+^ memory T cell subsets exhibit specific alterations in Gram-negative septic shock, which may hold significant prognostic value.

We found that non-survivors exhibited a significantly elevated proportion of CD8^+^ T_CM_ cells with an enhanced capacity to produce IL-2 at admission. This finding is consistent with the hypothesis that non-specific activation of CD8^+^ memory T cells may contribute to the early inflammatory response in sepsis (19–21). As the disease progressed, our study revealed that non-survivors had a significantly reduced proportion of CD8^+^ T_EM_ cells with markedly impaired abilities to produce TNF-α and IFN-γ by day 7. These findings are consistent with previous reports of impaired effector T cell function in severe sepsis (22–24). This likely forms a crucial immunological basis for the increased susceptibility to secondary infections and poor clinical outcomes observed in the later stages of septic shock (25, 26). Early activation of CD8^+^ T_CM_ cells and functional impairment of late CD8^+^ T_EM_ cells reveal cellular level changes in the dynamic immune microenvironment during septic shock (27–29). These temporally distinct alterations in CD8^+^ memory T cell subsets align with the well-recognized clinical trajectory of septic shock, where early inflammatory activation is often followed by suppressed immune function in critically ill patients. As an exploratory observational study, our findings cannot establish a causal relationship between these T cell phenotypic changes and the systemic pathophysiological shifts of the syndrome, but they provide preliminary cellular-level clues for further investigation into the immune dysregulation of septic shock.

Regarding clinical translation, our exploratory findings suggest that the proportion and activation status of CD8^+^ T_CM_ cells at admission are correlated with 28-day mortality. Immune monitoring focusing on memory T cell subsets may serve as a potential reference tool for clinical risk stratification in septic shock patients (30–32). Early identification of patients with specific immune phenotypes could facilitate more precise clinical interventions (33).

In addition, this study has several limitations. First, the sample size of this single-center exploratory study is relatively limited, which may restrict the reliability and generalizability of the findings. Multivariate adjustment for confounding factors such as SOFA score and lactate level cannot be performed, and conclusions regarding prognostic biomarkers should be interpreted with caution. Second, the study only enrolled patients with Gram-negative septic shock, and the findings may not be extrapolated to septic shock caused by other pathogens. Finally, this study is limited to phenotypic and functional observations, and the underlying molecular mechanisms warrant further investigation. In future research, further large-scale multicenter cohort studies are needed to validate the prognostic value of CD8^+^ memory T cell parameters, expand the range of pathogens included, and explore the specific molecular mechanisms of immune dysfunction in septic shock.

## 5 Conclusion

This prospective exploratory study reveals that Gram-negative septic shock presents parallel temporal aberrations in two distinct CD8^+^ memory T cell subsets: early expansion and activation of T_CM_ cells, and late depletion and functional impairment of T_EM_ cells. In this exploratory cohort, admission CD8^+^ T_CM_ proportion and IL-2 production capacity are associated with 28-day mortality of patients with Gram-negative septic shock, showing preliminary potential as candidate immune indicators for early risk stratification. Limited by the sample size, these findings need to be further verified in large multicenter cohorts. These findings provide new insights into the adaptive immune pathogenesis of sepsis and lay a foundation for the development of memory T cell-targeted immunotherapies.

## Data Availability

The raw data that support the findings of this study are available from the corresponding author upon reasonable request.
