# Supplementary material for: Immunological dynamics of memory T cells in gram-negative septic shock patients: a prospective clinical study
Source: Front Immunol. 2026 Sep 17;17:1923966. doi: 10.3389/fimmu.2026.1923966 (PMC13627360; doi:10.3389/fimmu.2026.1923966)
Supplement: Supplementary file 1 [file SupplementaryFile1.docx]

**Supporting Information**

**Immunological Dynamics of Memory T Cells in Gram-Negative Septic Shock Patients: A Prospective Clinical Study**

*Frontiers in Immunology*

Shuhang Wang^1✝^,Li Liu^1✝^, Ruichen Mao^1^,

Yanfen Chai^1*^, Yancun Liu^1*^

^1^ *Tianjin Medical University General Hospital, Tianjin 300052, China*

Number of Pages: 3

Number of Figures: 2

**Fig. S1.** (a) Hierarchical gating strategy for identifying CD4^+^ memory T cell subsets; (b) Dynamic changes in CD4^+^ memory T cell subsets between survivors and non-survivors of septic shock.

**Fig. S2.** Plasma cytokine concentrations in patients with Gram-negative septic shock measured by ELISA. (A) Comparison of plasma IL-2 level between survivors and non-survivors at admission. (B) Comparison of plasma IFN-γ levels between survivors and non-survivors on day 7 after admission. (C) Comparison of plasma TNF-α levels between survivors and non-survivors on day 7 after admission. ***P < 0.001, ns: not statistically significant.
